# Prevention of Diabetes after Gestational Diabetes: Better Translation of Nutrition and Lifestyle Messages Needed

**DOI:** 10.3390/healthcare2040468

**Published:** 2014-11-21

**Authors:** Sharleen L. O’Reilly

**Affiliations:** Centre for Physical Activity and Nutrition Research, Deakin University, 221 Burwood Highway, Burwood, Victoria 3125, Australia; E-Mail: sharleen.oreilly@deakin.edu.au; Tel.: +61-3-9244-6778; Fax: +61-3-9244-4219

**Keywords:** gestational diabetes mellitus, lactation, postpartum, lifestyle modification, type 2 diabetes mellitus, diabetes prevention

## Abstract

Type 2 Diabetes Mellitus (T2DM) and Gestational Diabetes (GDM) are important and escalating problems worldwide. GDM increases the risk of complications in pregnancy and birth, as well as a 1 in 2 chance of developing T2DM later in life. The burden of GDM extends to offspring, who have an increased risk of obesity and diabetes—further perpetuating the cycle of diabetes within families. Clinical trial evidence demonstrates T2DM incidence reduced by up to 50% for women with GDM with nutrition and physical activity changes and the economic modeling suggests cost effectiveness. The key diet-related changes to reduce T2DM risk are reviewed, in addition to breastfeeding. The difficulties associated with the delivery of dietary and lifestyle behaviour change to women after GDM are discussed and focus on: complex healthcare system interactions needed for care delivery; women finding postpartum self-care challenging; and low levels of awareness being present across the board. In addition, studies currently underway to improve care provision in this important area will be examined.

## 1. Introduction

The International Diabetes Federation (IDF) estimated that in 2013, 21.4 million or 16.8% of live births to women had “some form of hyperglycaemia in pregnancy” [1]. This is in the setting of rapidly increasing numbers of people diagnosed as having diabetes, and a projected rise of 55% by 2035 [1]. Diagnosed gestational diabetes (GDM) affects approximately 7% of pregnancies worldwide [2]. Furthermore, GDM incidence rates are increasing attributed mainly to rising population obesity levels [2]. In Australia by 2023, diabetes is estimated to be the largest contributor to the burden of disease [3] and by 2033, the economic burden is estimated to be $7 billion [4].

At least 17,000 Australian women are diagnosed with GDM every year [5] but this is most likely an under representation. Bullard *et al.* reported that the prevalence of prediabetes defined by fasting plasma glucose (FPG) and glycated haemoglobin A1c (HbA1c) in the US for their 18–44 year olds was 26% [6]. Extrapolating from the Hyperglycaemia and Adverse Pregnancy Outcome study, the overall rate of GDM was 16% and 13% in the two Australian Field Centres, Brisbane and Newcastle [7]. Hence, these data suggest that diagnostic rates less than 10% are likely to be missing women (and their offspring) at significant risk of adverse pregnancy outcomes due to hyperglycaemia. Applying the new International Alliance of Diabetes in Pregnancy Society Guidelines/World Health Organisation diagnostic criteria [8,9] to the most recent Australian census data (in 2011 there were 301,620 live births), over 54,000 women—1 in 6 pregnancies—would be identified with GDM annually. While this figure on its own presents an enormous challenge for maternity services, it is evident that it will also have substantial knock-on increases in pathology testing and further strain on the already constrained primary care sector.

GDM is the single strongest population predictor of T2DM development and there is at least a seven fold increase in T2DM in women who have had GDM compared to those with euglycemic pregnancies [10]. Those who have had GDM during one pregnancy have 30%–50% risk of developing GDM in another pregnancy [11,12] and the GDM pregnancy and birth itself will have an increased risk of complications for both mother and baby [13]. Moreover, women with a history of GDM are at increased risk of cardiovascular disease (CVD) [13,14] and the burden of GDM extends to their offspring, who themselves have an increased risk of obesity and diabetes [15,16]—further perpetuating and potentially expanding the cycle of diabetes within families.

In the face of this growing tide of T2DM coming from women who have had GDM, a healthcare chasm has begun to surface because the majority of women who have had GDM are not being screened regularly [17,18] nor receiving consistent diabetes prevention care [11,19,20]. This chasm has been subject to a recent call to action by the National Diabetes Education Program and American College of Obstetricians and Gynecologists, asking primary care providers to better meet the needs of this group of women [21]. Taking the call to action on-board along with a recent Australian call [22], this review will focus on the primary care sector and examine the issues surrounding current best practice guidelines for postpartum diabetes prevention. The review will investigate the core behaviour change areas within the guidelines, namely lifestyle modification and the importance of breastfeeding. In addition, it will look at new studies currently underway that seek to translate the evidence into improved provision of care to this population.

## 2. Best Practice Guidelines for Diabetes Prevention in Women Who Have Had GDM

Evidence-based care requires evidence-based intervention [23], which necessitates strategies that improve the uptake and use of clinical practice guidelines by the intended guideline audience [24]. Recommendations for the prevention of T2DM within different population subgroups exist around the world. They are typically the result of inputs from professional bodies and organisations, where they seek to digest the available evidence into a format that will result in improved quality of care after careful consideration of the strength and applicability of research findings. However, a common problem with best practice guidelines is that several exist for a single health condition or care delivery setting which can result in healthcare practitioner inactivity due to the lack of consistent guidance.

There are three main Australian guidelines circulating for the follow-up of women at risk of diabetes (Australian Diabetes in Pregnancy Society (ADIPS) [25], Therapeutic Guidelines Limited [26] and Guidelines for Preventative Activities in General Practice [27]) but unfortunately they lack a consistent approach for GDM follow-up care (Table 1). All three guidelines are inconsistent with the current NICE and American Diabetes Association guidelines [28,29] so while GPs recognise the importance of follow-up [19] and the variety of professionally endorsed options arguably gives them a broader set of clinically valid alternatives, the level of choice may paradoxically leave them feeling more exposed to risk [30,31]. In a recent Australian study, GPs involved in shared maternity care reported awareness and usage of a wide variety of guidelines [32]. While these GPs are not representative of the broader GP community and as a group their care provision should be a model of best practice, even this group was not adhering to the lifestyle modification guidance [32]. A large UK survey of primary and secondary care providers reported the sole national NICE guideline was not consistently being adhered to [33] so while having several guidelines is a possible issue, it is certainly not the only barrier to better care provision.

A diagnosis of GDM will typically result in a mother being defined as a higher risk pregnancy and as such, her care will be moved from any GP or midwife care to a specialist consultant and hospital-based care arrangement. However, once a mother is discharged from maternity services, the continuity of her care can be disrupted particularly in relation to her GP-led postnatal GDM care. Health care providers typically report issues around these women being lost to follow up and a lack of continuity of care existing [20,33,34] and this sentiment is also echoed in women [20,35,36]. Research into improving continuity of care is challenging because of the different healthcare systems and how they operate. One systems level change initiated is the Australian National Gestational Diabetes Register (commenced July 2011), the first national registry of women with GDM that works across healthcare systems. A woman’s registration commences in pregnancy and the registry delivers annual reminders to them and their health care providers about the importance of screening and a healthy lifestyle [37].

**Table 1:** Guidelines for postnatal diabetes prevention care of women who have had gestational diabetes.

Guideline Topic	RACGP [27]	TG Ltd. [26]	ADIPS [25]	NICE [28]	ADA [29]
**Postnatal screening**	6–12 weeks after delivery; OGTT	6–12 weeks after delivery; 75 g OGTT	6–12 weeks after delivery; OGTT	6 weeks after delivery; FPG	6–12 weeks after delivery; 75 g OGTT
**Repeat screening**	3 Yearly	Yearly (Alternatively 75 g OGTT every 2 years or if contemplating further pregnancy)	Yearly; OGTT if contemplating further pregnancy	Yearly	1–3 Yearly; yearly if IFG or IGT, otherwise 3 yearly
**Repeat screening test**	FPG	FBG or RBG	75 g OGTT or FPG	FPG	75 g OGTT
**Lifestyle recommendations**	General healthy eating Increase physical activity (30 min brisk walking 5 days a week) and/or weight loss. Encourage breastfeeding	Healthy diet and exercise	Weight control Healthy diet and exercise	Weight control Healthy diet and exercise Encourage breastfeeding	Weight loss 7% body weight Low fat Increase fibre at 14 g/1000 kcal and whole grains Increased PA to 150 min/week moderate activity

IFG = Impaired Fasting Glucose, IGT = Impaired Glucose Tolerance, FPG = Fasting Plasma Glucose, FBG = Fasting Blood Glucose, RBG = Random Blood Glucose, OGTT = Oral Glucose Tolerance Test.

### Postpartum Screening for Type 2 Diabetes

T2DM screening rates are low for women with a history of GDM [34,38] and there are several reasons why this is occurring. Recent Australia data for Queensland shows the estimated probability of women receiving any diabetes screening test within the first three years postpartum to be 49% and this drops to 34% for the oral glucose tolerance test (OGTT) [17]. Early infancy is a busy time for mothers—their own health tends to be neglected—with women citing time pressures, dislike of testing procedures, inconvenience and lack of childcare as barriers to screening [19,20,38,39]. General practitioners on the other hand report being undecided on which blood test to use [11,12,20,40]. Several guidelines recommend fasting blood glucose because the gold standard OGTT is so time-consuming, yet other guidelines recommend it [25,26,27,41]. A similar predicament is presented to GPs when they seek to find out what is the ideal time point to repeat a woman’s screening test—spacing varies from one to three years [25,26,27,41]. An additional complexity surrounds the use of HbA1c to diagnose diabetes, HbA1c is recommended as a suitable test to diagnose diabetes [29] but the guidelines for women who have had GDM do not yet endorse it even though HbA1c would present a much more appealing annual screening option for busy mothers as it is a non-fasting test [25,42]. The barrier in Australia to HbA1c being used is that currently reimbursement by Medicare can only happen when the person has diagnosed diabetes [43]. Given the spread of these practical issues, it is not surprising that systematic screening is not happening [22,32].

**Figure 1:**
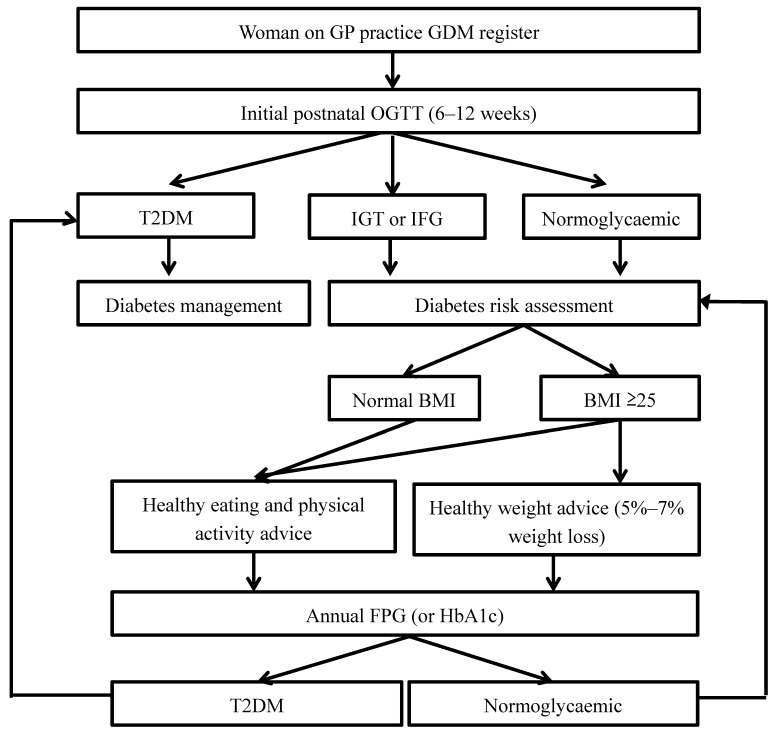
Postnatal management for women who have had gestational diabetes. Simplified care plan within a flowchart for a woman with a Gestational Diabetes (GDM) pregnancy based on guidelines. The creation of a register within a GP practice will enable recall and reminders at appropriate time points. Abbreviations: HbA1c (glycated haemoglobin A1c); GP (general practitioner); GDM (gestational diabetes); OGTT (oral glucose tolerance test); IGT (impaired glucose tolerance); IFG (impaired fasting glucose); BMI (body mass index); FPG (fasting plasma glucose); and T2DM (type 2 diabetes).

Attendance rates for screening are consistently low for women with a history of GDM, ranging from 35% to 56% [10,11,34,44]. Four potential strategies have been put forward to increase the rates of screening amongst women with a history of gestational diabetes based on the evidence: simplify testing [18,38]; increase awareness across the board [19,38,45]; improve organisation of care [40]; and set-up automatic reminders (GPs and women) [18,19,46,47,48]. While all of the strategies have been used to good effect in women postnatally, the main problem is delivering them together in a coordinated manner within primary care settings [20,22,49]. A simplified care plan (Figure 1) has been developed for women with GDM, the flowchart starts with the initial postnatal period and incorporates several of the strategies mentioned above into the care process and uses guideline-led care to map out what a more coordinated approach might look like in practice.

## 3. Type 2 Diabetes Prevention

Clinical trials demonstrate T2DM incidence reduced by up to 58% for high risk people following lifestyle change [50,51,52] and economic modeling suggests lifestyle modification programs are cost saving or very cost effective [53,54]. The US Diabetes Prevention Program (DPP) provided evidence that lifestyle change works for women post-GDM, their T2DM risk reduced by 50% [55] and the effect of diabetes prevention continued 10 years after the intervention within the whole DPP cohort [56]. All three large lifestyle modification studies have been able to show sustained reduction in conversion to T2DM (though not specifically in women who have had GDM), ranging from 34% at 10 years [56] through to 43% at 7 years [57] and 20 years [58]. Therefore, addressing GDM constitutes a window of opportunity for early intervention and reduction of the future burden of T2DM.

DPPs have traditionally been designed to meet the needs of an older population at risk of developing diabetes, the mainstay of clients attending programs. However, women who have had GDM are younger and will typically have different life stage issues as a result. Those differences become important when considering the design and delivery of a DPP for this population. Looking at qualitative work in this area shows that issues include: provision of childcare for attendance; accessibility of the venue (feeding facilities and pram access); fitting DPP attendance into busy family life; needing a family-friendly lifestyle modification; and affordability of the DPP [20,59,60,61,62]. There is currently a large Canadian prospective cohort study seeking to further elucidate this within a multiethnic group [63] as the previous work has been within small groups of women and it is important to know which barriers hold across the broader GDM population and are important considerations for larger scale DPPs.

### 3.1. Risk Awareness

Perception of risk is important to motivate women for both follow-up testing and uptake of lifestyle modification messages [64]. Despite understanding the association between GDM and T2DM, women with GDM do not perceive themselves to be at elevated risk [19,36,64]. The lack of perceived self-risk of developing T2DM is common in women [34,64,65] and awareness amongst health professionals and the general public of GDM’s risk is also low [19,66]. A systematic review of diabetes care in the primary care setting found that health outcomes improve for consumers if personalised risk communication and communication before consultations are provided [67]. FINDRISC [68] and AUSDRISK [69] are examples of tools that could potentially be used to initiate risk communications within the primary care setting for women with a history of GDM.

### 3.2. Lifestyle Modification

The evidence around lifestyle modification shows a definite and maintained decrease in progression to T2DM in a variety of populations [50,52,55,70,71,72,73]. While there is uniform agreement that lifestyle modification is warranted to prevent diabetes, the way in which that message is communicated varies between guidelines (Table 1) leaving room for difference in interpretation by caregivers and the women themselves [20]. The guidelines for physical activity are relatively consistent but it is the area of nutrition where difference is more evident.

Women of childbearing age will typically see their GP 12 times in a year—making them the healthcare professional most regularly contacted during that period [74]—and a logical setting for care provision and/or coordination. GPs are also the main source of nutrition care in the primary care setting—discussing nutrition concepts in ~7% of consultations—equating to over 7.9 million occurrences per year in Australia [75]. This finding is likely to be more reflective of limited access to dietitians, with expertise in the delivery of lifestyle modification, rather than patient preference due to issues such as distance, cost, lack of awareness or availability affecting choice [76]. Within the above context, the GP and practice staff become the resource that women will typically use to support their engagement in diabetes prevention, alongside any available community-based public health programs [77]. Systematic review validates GP capacity to provide nutrition care that enhances nutrition behaviour and risk factors in individuals with lifestyle-related chronic disease [78] and that advice from GPs can be a powerful motivator for women to adopt lifestyle modification [79,80]. However, the evidence points to GPs needing more training in the delivery of lifestyle advice—particularly nutrition advice—for diabetes prevention [20,81,82]. Shortfalls in nutrition knowledge are the likely reason for GPs stating reduced confidence and self-perceived ability in delivering nutrition care [81,83], even though they report strong positive attitudes on the importance of providing nutrition care to patients with chronic disease [81,82]. Evidence for the effectiveness of nutrition care in general practice, particularly in relation to GDM, is unclear as a result and indicates that further support is needed for GPs to provide diabetes prevention-relevant lifestyle advice to patients [20,84] and that GPs will typically be more comfortable coordinating a women with a history of GDM’s care and monitoring her health markers than delivering specific lifestyle advice [32].

#### 3.2.1. Weight Loss

Weight loss is thought to be the risk factor that explains the greatest variance in risk and the one that is most amenable to change [85]. Maternal pre-conception weight has been increasing in line with population gains [86]. We know that in a large recent study of over 22,000 women, mothers who gained over 3 kg/m^2^ between pregnancies had 3.4 times higher odds of developing T2DM [87]. Even a 1–2 kg/m^2^ maternal gain resulted in 1.7 raised T2DM odds but the positive message was, mothers who were overweight at their index pregnancy and subsequently lost around 2 kg/m^2^ decreased their risk by almost 80% [87]. It is not simply weight gain after delivery that is clinically relevant but also weight gain during and after pregnancy [88]. Peters *et al.* demonstrated that for every 4.5 kg weight gained after delivery there was a doubling in risk for T2DM development, even after adjustment for OGTT results, post natal BMI and breastfeeding [89].

As with all lifestyle interventions, successful weight loss is difficult to achieve. The Diabetes Prevention Program’s GDM arm achieved 7% weight loss, which resulted in a 53% reduction in T2DM incidence [55]. However, the mean weight loss of women with a history of GDM was only 1.6 kg at 3 years (3.5 kg regain) compared to 4 kg mean weight loss for women in the DPP without GDM [55]. Within diabetes prevention studies the relative risk reduction for T2DM development is 16% for each kilogram lost [90] but weight regain is also well documented [56] and thought to happen due to the body’s physiologic drive to return to the original higher weight [91] and a whole raft of psychosocial barriers [20]. However, a simple effective tool in supporting weight management and long-term weight loss is self-monitoring [92,93,94] and this can be readily incorporated into the care of women with a history of GDM.

#### 3.2.2. Macronutrients

Apart from weight loss, the composition of the diet is important in GDM and T2DM prevention. Within the Diabetes Prevention Program, the reduction of energy from fat and increased levels of physical activity were predictive and contributed to sustained weight loss [90]. The Finnish Diabetes Prevention Study had additional dietary targets outside weight loss and increased physical activity from the Diabetes Prevention Program that were: less than 30% energy from fat; less than 10% energy from saturated fat; and more than 15 g dietary fibre per 1000 kcal [95]. While the only significant association for diabetes risk in the intervention was weight loss, the authors proposed the dietary composition and physical activity were important but mediated their effects through weight reduction [57].

Dietary patterns that align with the Finnish Diabetes Prevention Study dietary targets have been associated with decreased risk of diabetes among women who have had gestational diabetes, although such studies are few. A Korean study looked at dietary composition postnatally (6–12 weeks) and found a greater saturated fat intake and a higher ratio of fat to total energy was associated with diabetes and pre-diabetes [96].

The Nurses’ Health Study II cohort is the main dataset used for prospective studies exploring associations in women and pregnancy. Dietary fibre and glycaemic index were explored for associations with GDM occurrence and the main outcome was that for every 10 g/day total fibre increase there was a 26% reduction in GDM risk (95% CI 9–49) [97]. In a recent Nurses Health Study cohort, 4413 women with histories of GDM were followed over 14 years and had their dietary patterns assessed at 4 yearly intervals; women’s dietary patterns were scored by several scales based on the Mediterranean diet, Dietary Approaches to Stop Hypertension (DASH) diet and the Healthy Eating Index [98]. Women consuming diets within the highest quartile for the Mediterranean diet scale had 40% lower T2DM risk compared with those in the lowest quartile (hazard ratio 0.60, 95% CI 0.44–0.82). Similar risk reductions were seen for the DASH diet (46% lower risk, hazard ratio 0.54, 95% CI 0.39–0.73) and Healthy Eating Index (57% lower risk, hazard ratio 0.43, 95% CI 0.31–0.59) [98] even after multiple adjustments (age, total calorie intake, age at first birth, parity, ethnicity, parental diabetes, oral contraceptive use, menopause, and smoking) but the risk reduction was partially mediated by body mass changes. In a subsequent study, 21,411 singleton pregnancies were identified prospectively over a 10 year period and low carbohydrate dietary patterns analysed against GDM development [99]. Women with a pre-pregnancy low-carbohydrate dietary pattern with high animal food sources of saturated fat and protein and fat from animal-food sources were at significantly more risk of GDM development but those with a low-carbohydrate and high vegetable food sources were not at a raised risk [99].

#### 3.2.3. Physical Activity

The message on physical activity within guidelines for women with a history of GDM is relatively consistent. However, the evidence to support the benefit of physical activity in preventing diabetes in these women comes mainly from the China Diabetes Prevention Study [52] and there has been some doubt cast over the quality of the data [100]. However, Bao *et al.* recently showed women who have had a GDM pregnancy within the Nurses Health Study II cohort had a 9% risk reduction for T2DM development (adjusted relative risk 0.91; 95% CI, 0.88–0.94) for every 100 minutes of moderate intensity physical activity they had, which remained significant after BMI adjustment. Importantly, women who increased their physical activity by 150 minutes moderate intensity per week compared to those who maintained their physical activity level were 47% less likely to develop T2DM (RR, 0.53; 95% CI, 0.38–0.75); again the association remained significant after BMI adjustment [101]. Conversely, high levels of sedentary behaviours such as screen time were associated with increased risk [101] making both sides of the physical activity equation important considerations.

### 3.3. Breastfeeding

Breastfeeding is known to provide numerous health benefits to both mother and baby. For mothers who have a history of GDM, improved lipid and glucose metabolic profiles during the first 3 months following delivery are reported but the downside is that they breastfeed for a shorter length of time and are less likely to start breastfeeding in the first place [102]. Longer term good quality studies found breastfeeding for >3 months decreased the risk of T2DM by >40% (HR 0.54, 95% CI 0.34–0.85) after adjusting for islet autoantibodies, family history of diabetes, maternal BMI, age, and smoking [103] and breastfeeding for >10 months improved glucose tolerance, and increased insulin sensitivity and insulin secretion/insulin sensitivity [104]. Ziegler *et al.* also found in their prospective study that breastfeeding for ≥3 months delayed T2DM onset by 10 years compared with <3 months breastfeeding [103]. It is also worth noting that the reduction in risk did not appear to be entirely mediated through weight because change in weight postnatally and length of breastfeeding were not significant [103].

The awareness levels of mothers around the benefits of breastfeeding for their babies is generally high, however the benefits to their own long-term health is frequently overlooked when they are receiving education. It is now apparent that breastfeeding can prevent metabolic syndrome [105] and preserve beta cell function [104] as well as improved postnatal weight loss, reduced obesity [102] and T2DM risk [103]. A mother’s decision to commence and continue to breastfeed is normally based on the benefits her baby will receive but it is essential for women who have had GDM to receive education from their healthcare providers on the health benefits to their own health and receive adequate breastfeeding support [102]. Considering the typical demographic of women with GDM coming from lower socioeconomic backgrounds, breastfeeding offers a safe and low-cost intervention to prevent T2DM development.

Successful breastfeeding is dependent on effective lactogenesis occurring during both lactation stages (stage 1: early colostrum production and stage 2: increased milk volume production between 36 h and 92 h post-partum). Matias *et al*. recently reported a third of women with GDM in a prospective cohort study having delayed onset of stage 2 lactogenesis [106], the risk factors associated with that delay being maternal obesity (OR 1.56, 1.07–2.29 95% CI), insulin treatment (OR 3.11, 1.37–7.05 95% CI) and suboptimal in-hospital breastfeeding support (OR 1.65, 1.20–2.26 95% CI) [106]. The associations between maternal obesity and lower rates of breastfeeding initiation are supported by other studies [107,108] and while breastfeeding initiation is not normally managed in primary care, incorporating advice into pre-conception counseling would be potentially beneficial for women.

## 4. Intervention Programs

With the increasing recognition of the importance of postnatal care in women who have had GDM [21,22], the level of research interest in improving the quality of that care has grown. High quality randomised controlled trial (RCT) data are needed to understand what constitutes an effective DPP in this population. There are several studies currently underway with the aim of developing feasible and effective programs to reduce T2DM risk in this population (Table 2). While not an intervention study, information on the impact of health beliefs, social support and self-efficacy on postnatal physical activity and dietary habits of women with GDM [109] will be reported in 2015. The study should allow development of a predictive theoretical model to optimise interventions in women who have had GDM, as it will be following a large cohort of 200 women prospectively and build on information gleamed from smaller qualitative datasets.

The OGTT, as discussed previously, is a problematic test within this population but the clinical benefit of using it in women who have had GDM means that alternative strategies need to be investigated to improve compliance so as to maximize screening rates. The DIAMIND study is an RCT being trialed to improve screening rates through the use of SMS text message reminders [110] It plans to build on the evidence that reminders are an effective tool in increasing screening rates [20] and will use SMS’s at 6 weeks, 3 and 6 months to encourage OGTT testing during the first 6 months post-partum *versus* SMS reminder only at 6 months in the control group. The outcome measure for DIAMIND will be the difference in OGTT screening rates between groups.

Debate exists about the most effective time point to intervene with a DPP in this population and as a result, interventions registered have either a postnatal-only [111,112,113,114] or a mixed approach that includes pregnancy and the postnatal period [115,116,117,118]). The mixed approach proposes that commencing education while a mother is pregnant builds on the healthy habits developed through intensive management of her GDM and a mother’s motivation to look after the wellbeing of her infant [115]. However, the postnatal only approach would argue that it can be problematic to translate into practice where distinct divisions between maternity and primary care services exist [119,120] and that women report the postnatal period to be a better time to start a DPP (where the focus is on their own health, not that of their infant) [62]. It remains to be seen which time point is more effective but the outcomes will most likely point to a tailored DPP approach for individual healthcare systems being the most beneficial.

The results from DPP interventions have been few to date. Dulce Mothers reported earlier this year [113] on a translated US DPP for low income Latina women with a history of GDM. The study had a pre-post test design for the 8-week peer-led group education program. Dulce Mothers found a significant decrease in total cholesterol, LDL-cholesterol and triglycerides for the intervention but no change in body weight and a significant increase in HbA1c [113]. The lack of a control arm alongside the short 3-month follow-up period and some methodological concerns effectively limits the conclusions that can be drawn from the study. A pilot RCT of a phone-based lifestyle education program using motivational interviewing of 38 Australian women demonstrated significant changes to total fat intake, leisure time physical activity and body mass index but this was also limited by the sample size [121]. The DEBI study was a pilot RCT that reported early promising results [122,123] and it has subsequently been scaled up to a larger RCT intervention, the GEM study [115]. DEBI randomized 197 women to receive a telephone and face-to-face delivered DPP with additional breastfeeding support during the woman’s pregnancy and postnatally *versus* usual care. The pilot found a significant decrease in dietary fat (−3.6%, *p* = 0.002) and changes that were close to significant for weight loss and increased breastfeeding [123] and a sub-group analysis showed women who lost >2 kg had improved glucose metabolism over those who maintained or gained weight [122].

Within the interventions where results are pending (Table 2), the key variance appears to be the level of intensity of contact with women and the mode of contact. The majority of the studies taking an individual approach are intensive and require around 13–20 contact sessions with each woman [115,117,123,124]. The lower end of the contact spectrum (~6 contacts) tend to use group settings to bring in peer interactions, which is a known enabler to change in this population [20] and a core component of the main DPPs [77]. The modes of contact typically involve face-to-face contact but some interventions are augmenting this traditional mode with SMS texts [110,116], telephone and/or print communications [111,112,115,117] as a way of reducing costs but also reflecting the common barrier reported by mothers of insufficient time to engage in behaviour change [20,62]. There are several interventions that plan on examining health economic and fidelity measures within the RCT [111,112,115,125] and these will be critical in the research translation process as they will enable greater uptake of the findings into areas like policy and practice due to their external validity. The ability to analyse the findings of the interventions within a systematic review and suitable meta-analysis will give healthcare providers and policy makers valuable information about effective ways to work with women who have had GDM to make lifestyle modifications and reduce their risk of T2DM.

**Table 2:** Registered lifestyle interventions to reduce type 2 diabetes risk in women who have had gestational diabetes.

Study Title	Recruitment Target	Intervention	Follow Up Duration	Primary Outcome Measures	Estimated Completion
**Mothers After Gestational Diabetes in Australia study (MAGDA) [111,112]**	574 Australian women (Victoria and South Australia)	1 individual session and 5 group sessions (initial 3 months) and 2 follow-up telephone sessions. 5 goals: 5% weight reduction, <30% energy from fat, <10% energy from saturated fat, 15 g fibre/ 1000 kcal, >30 min daily moderate or vigorous exercise.	1 year	Diabetes risk (FPG, weight or waist circumference)	2015
**Tianjin Gestational Diabetes Prevention Program (TGDPP) [114]**	1180 Chinese women (Tianjin province)	5 individual consults with dietitian (year 1) and 2 individual consults with dietitian (year 2). 6 goals: 5%–10% weight reduction in overweight women through > 10% reduction in total energy, <30% energy from fat, <10% energy from saturated fat, 55%–65% energy from carbohydrate, 20–30 g per day fibre, >30 min daily moderate or vigorous exercise.	2 years	Incident T2DM	2013–2014
**The DIAMIND study [110]**	276 Australian women (South Australia)	SMS text OGTT reminder at 6 weeks postpartum, with further reminders at 3 and 6 months if not tested. Control group receive single SMS text reminder at 6 months postpartum.	6 months	OGTT attendance by 6 months post-partum	2014
**Lifestyle Intervention Program for Women With Gestational Diabetes or Gestational Impaired Glucose Tolerance (APPLES) [124]**	350 American women (California)	1 individual session and 3 telephone counseling sessions (Phase 1, during pregnancy). After 6 weeks post-partum, 3 individual sessions and 13 telephone counseling sessions (phase 2, 6 months). DPP goals (weight reduction and increased physical activity).	2 years	Achievement of pre-pregnancy weight if normal BMI pre-pregnancy or 5% weight reduction on pre-pregnancy weight if overweight	2016
**Croí MyAction program [125]**	54 Irish women (Galway)	1 individual risk assessment and 2.5 h session weekly for 12 weeks (1 h group exercise, 1 h group education and 30min individual session). Support person participates in 12-week program. Goals: BMI >30 kg/m^2^, >30 minutes moderate intensity physical activity >5 days per week, Mediterranean dietary pattern.	1 year	Mean FPG reduction on OGTT	2014
**Optimizing Outcomes in Women with Gestational Diabetes Mellitus and their Infants study [116]**	100 American women (North Carolina)	Phase 1 (4 months): 1 group session (during pregnancy) and 13 group sessions (post-partum, includes 1 h exercise class). Phase 2: 3 group sessions at monthly intervals. Weekly SMS text from enrollment to study completion.	10 months	FBG and weight (BMI)	2014
**Dulce Mothers [113]**	84 Latina American women (California)	8 weekly 2 h group sessions including 15–20 minutes exercise. DPP goals (weight reduction and increased physical activity).	6 months	HbA1c, blood lipids and weight (BMI)	2014 (completed)
**Estudio Parto [117]**	300 Hispanic American women (Massachusetts)	Phase 1: 1 individual session (during pregnancy), 1 individual session (6 weeks post-partum), weekly/fortnightly/monthly print and telephone contact (6 months). Phase 2: monthly/bimonthly print and telephone contact (6 months)	1 year	Weight (BMI), insulin resistance markers and cardiovascular disease risk markers	2016
**Gestational Diabetes’ Effects on Moms (GEM) study [115]**	2320 American women (California)	Phase 1: Individual weight goal letter (during pregnancy). Phase 2: 13 telephone sessions and support materials (6 months). Phase 3: 3 newsletters and support materials (6 months)	1 year	Achieving post-partum weight goal and total weight change	2014
**Prevention of gestational diabetes through lifestyle modification (RADIEL) study [118]**	728 Finnish women (South Eastern region)	Individual counseling 3 monthly pre and during pregnancy and at 6 weeks, 6 months and 12 months. Goals: 5%–10% weight loss pre-pregnancy if BMI ≥25 kg/m^2^ or no weight gain during first 2 trimesters for pre-pregnancy BMI ≥30 kg/m^2^, >30 min daily moderate or vigorous exercise, 1600–1800 kcal/day, 40%–50% energy from carbohydrates, 30%–40% from fats and 20%–25% from protein.	1 year	OGTT	2014

## 5. Conclusions

Gestational diabetes presents us with a tantalising challenge. We know women who develop GDM are at high risk of developing T2DM, yet what remains outside our grasp is how to effectively translate the prevention information into practice. The screening process enables us to identify women at risk and start prevention measures earlier but there are issues surrounding engaging women and their healthcare providers such as GPs in the process over time. There is recognition amongst professional representative bodies that diabetes prevention within this sub-group of the population is important. However, the variance between guidelines from these bodies is not conducive to a universal approach to care delivery.

The translation of the key lifestyle components into effective evidence-based practice needs to overcome numerous implementation barriers. The proposed solutions to those barriers being: effective prevention program development; evidence-based care delivery by GPs; system level change to improve transfer and monitoring of medical information; awareness raising of GDM as a diabetes predictor; alongside environmental and social supports to promote healthy eating and physical activity. If those implementation dominos were to line up, what we would see would be an evidence-to-practice bridge being formed with the risk of diabetes fading for women who have had GDM and a healthier future for them and their families. However, there is still plenty of work to be done and the outcomes of several forthcoming intervention studies should help shine some light on the paths we need to follow.
